# Impaired immune surveillance of senescent hepatocytes in MASLD: SASP signaling and fibrogenic progression

**DOI:** 10.3389/fimmu.2026.1982821

**Published:** 2026-10-14

**Authors:** Shuhao Cheng, Yinan Zhao, Wei Wang

**Affiliations:** 1 Jiaozuo Second People’s Hospital, Jiaozuo, China; 2 Clinical Medical College of Qinghai University, Xining, China

**Keywords:** hepatic stellate cells, hepatocyte senescence, immune surveillance, liver fibrosis, MASLD, SASP, senotherapy

## Abstract

Metabolic dysfunction-associated steatotic liver disease (MASLD) is sustained not only by lipid overload and hepatocellular injury but also by maladaptive responses to persistent metabolic stress. Cellular senescence has emerged as a candidate mechanism linking metabolic stress to persistent inflammation and fibrosis. This Mini Review aims to examine how hepatocyte senescence develops and persists in MASLD, how senescent hepatocytes interact with immune surveillance, and how senescence-associated secretory phenotype (SASP) signaling contributes to fibrotic remodeling. As a narrative review, we synthesized recent human, animal, and *in vitro* evidence, prioritizing mechanistic studies directly addressing hepatocyte senescence, immune-cell interactions, SASP signaling, and fibrosis, while retaining landmark studies where they provided essential mechanistic context. Current evidence indicates that senescent hepatocytes accumulate in experimental and human MASLD and can influence macrophages, hepatic stellate cells, liver sinusoidal endothelial cells, and neighboring hepatocytes. Under conditions of effective immune surveillance, senescence may remain transient and contribute to the removal of damaged cells. In chronically injured liver, however, continued generation of senescent hepatocytes, altered immune-cell function, and persistent SASP signaling may favor their retention and promote inflammatory and fibrogenic remodeling. Important uncertainties remain, including the lack of universal senescence markers, limited direct evidence for defective senescent-hepatocyte clearance in human MASLD, and the context-dependent effects of senescence across hepatic cell types. Overall, available evidence supports hepatocyte senescence as a plausible contributor to MASLD progression, but its pathogenic importance appears to depend on cellular context, immune competence, and the duration of the senescent state. Defining these conditions will be important for developing senescence-directed therapies without disrupting beneficial repair responses.

## Graphical Abstract

**Figure:**
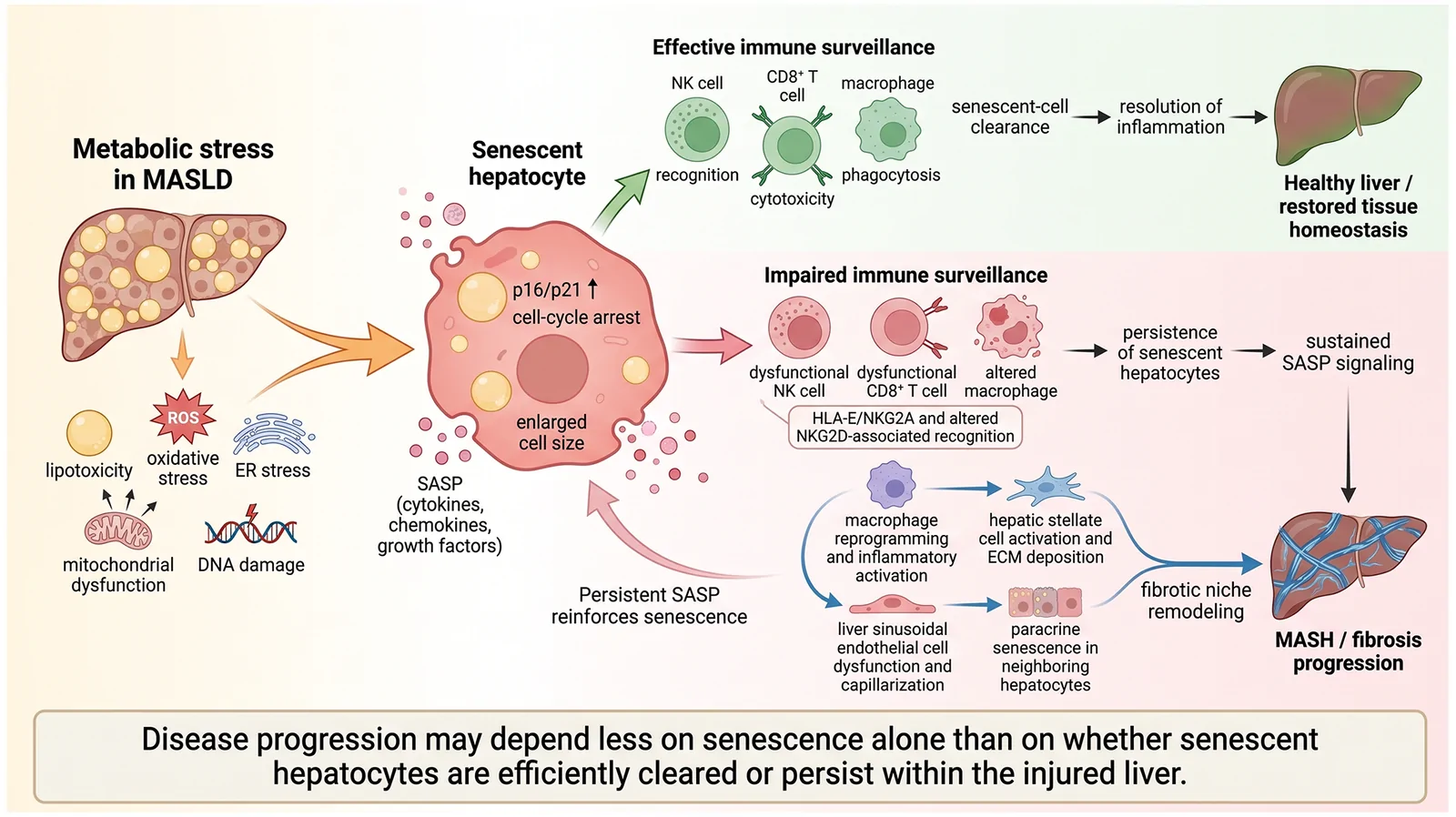
Created by BioRender (Agreement number: QT2A8GXA1F).

## 1 Introduction

Metabolic dysfunction-associated steatotic liver disease (MASLD) encompasses a spectrum ranging from isolated steatosis to metabolic dysfunction-associated steatohepatitis (MASH), fibrosis, and cirrhosis (1). Current estimates suggest that the disease affects approximately 38% of adults and 7–14% of children and adolescents worldwide (2, 3). Although excess lipid storage is a defining feature, disease progression is increasingly understood as a failure of hepatic adaptation to sustained metabolic stress rather than a simple consequence of steatosis (4). Lipotoxicity, oxidative injury, mitochondrial dysfunction, and impaired proteostasis repeatedly damage hepatocytes and promote inflammatory tissue remodeling (5–7). Yet the processes that allow injured cells to persist and convert a transient repair response into chronic fibrosis remain incompletely defined. Importantly, senescence is not the inevitable outcome of hepatocellular injury. Metabolically stressed hepatocytes may undergo reversible adaptation, cell death, or other stress responses; senescence should therefore be viewed as one potential cell fate that may complement, rather than replace, these established mechanisms of MASLD progression.

Cellular senescence offers a plausible link between metabolic injury and maladaptive repair (8). Senescent cells undergo prolonged cell-cycle arrest while remaining metabolically active and may acquire a heterogeneous senescence-associated secretory phenotype (SASP), comprising cytokines, chemokines, growth factors, proteases, and extracellular vesicles (8–10). Evidence for hepatocyte senescence in MASLD spans human tissue, experimental models, and molecular profiling. Human studies have reported increased hepatocyte p21 expression, telomere-associated DNA damage, and nuclear enlargement in association with more advanced fibrosis, while experimental studies have identified increased senescence-associated beta-galactosidase activity, cell-cycle inhibitor expression, and senescent-hepatocyte gene signatures (11–14). Functional studies further suggest that factors released by senescent hepatocytes can activate macrophages and hepatic stellate cells, impair liver sinusoidal endothelial cell differentiation, and transmit senescence-associated signals to neighboring hepatocytes (8, 15). Together, these observations support a biological association between hepatocyte senescence and MASLD severity, although they do not establish senescence as a uniform or dominant driver of disease progression.

Senescence is not intrinsically pathological (7). During acute injury, it may limit the expansion of damaged cells and facilitate their immune-mediated removal (16). Its consequences also depend on the affected hepatic cell type: whereas persistent hepatocyte senescence is generally associated with impaired metabolic function and profibrotic signaling, senescence of activated hepatic stellate cells can restrict their proliferation and extracellular matrix production in some injury models (17, 18). Persistent senescent-cell accumulation may arise, however, when cell production exceeds local clearance or when immune surveillance becomes ineffective (16). In this Review, “immune surveillance of senescent hepatocytes” refers to the recruitment, recognition, and elimination of senescent hepatocytes by innate and adaptive immune cells, particularly NK cells, T lymphocytes, monocytes, and macrophages, through chemotactic signals, stress-associated ligands, and activating or inhibitory receptor pathways. Adaptive immune cells, natural killer cells, and macrophages can participate in senescent-cell recognition and clearance in the liver, although direct evidence that this process fails in human MASLD remains limited (16). It also remains unclear which senescent hepatocyte states are preferentially retained, which ligand–receptor interactions determine immune recognition or escape, and when a potentially adaptive senescent response becomes persistently pathogenic. These unresolved questions constitute a central mechanistic gap in the current understanding of MASLD-associated hepatocyte senescence.

This Mini Review therefore focuses on three closely linked questions: how hepatocyte senescence develops in MASLD, how senescent hepatocytes are recognized or tolerated by the immune system, and how persistent SASP signaling contributes to fibrotic niche remodeling. The scope is deliberately centered on hepatocyte senescence and its interaction with immune surveillance; other forms of hepatocyte injury, systemic immune dysfunction, and senescence in non-parenchymal hepatic cells are discussed only where they provide mechanistic context or help distinguish cell-type-specific effects. We also consider the mechanistic and therapeutic implications of distinguishing transient, potentially adaptive senescence from persistent pathogenic states.

## 2 Hepatocyte senescence in MASLD

MASLD exposes hepatocytes to persistent metabolic and organelle stress rather than a single senescence-inducing insult (19). Excess saturated fatty acids and toxic lipid intermediates disrupt mitochondrial respiration, increase reactive oxygen species (ROS) production, trigger endoplasmic reticulum stress, and impair autophagic and lysosomal turnover (20). When this damage cannot be adequately repaired, hepatocytes may enter a prolonged state of cell-cycle arrest maintained principally by the tumor protein 53 (p53)/cyclin-dependent kinase inhibitor 1A (p21) pathway and the cyclin-dependent kinase inhibitor 2A (p16^INK4a^)–retinoblastoma protein (RB) pathway (20, 21). Although this response may initially prevent severely damaged cells from proliferating, its persistence can compromise the regenerative and metabolic capacity of the hepatic parenchyma (20). A remaining uncertainty is when a reversible or protective stress response becomes a durable senescent state, particularly because the same metabolic insults can also trigger adaptation, quiescence, or cell death. This distinction is important when interpreting senescence-associated changes during different stages of MASLD.

Evidence linking hepatocyte senescence to MASLD has been obtained from human liver tissue, experimental models, and *in vitro* studies. These sources provide different levels of inference: human tissue offers the strongest evidence for clinical association, animal models permit functional or interventional testing, and *in vitro* systems provide mechanistic support under controlled conditions. In liver biopsies, increased hepatocyte p21 expression, telomere-associated DNA damage, and nuclear enlargement have been associated with more advanced fibrosis and an increased likelihood of disease progression (14). These observations are therefore best interpreted as associations between senescence-related features and disease burden rather than direct evidence that any individual marker drives MASLD progression. In ageing, obese, and diabetic mice, senescence-associated changes occur alongside hepatic lipid accumulation, whereas genetic depletion of selected p16^INK4a^-expressing cells or pharmacological senolytic treatment reduced steatosis in some experimental settings (12). Exposure to free fatty acids also induces senescence-like changes in cultured hepatocytes, including oxidative stress, increased senescence-associated beta-galactosidase (SA-β-gal) activity, elevated cell-cycle inhibitor expression, and reduced proliferative capacity (22). More recently, one study reported that a senescent hepatocyte gene signature generated from complementary experimental systems was found to be enriched in human MASLD datasets, to increase with disease severity, and to decline during disease regression (13). Because this association was derived from a single recent study, the signature should currently be regarded primarily as a marker of senescent-hepatocyte burden that tracks with disease activity rather than as evidence that the signature itself represents a causal disease driver. These observations support the presence of clinically relevant but molecularly heterogeneous senescent hepatocyte populations (13).

Polyploidy adds another layer of complexity to the interpretation of hepatocyte senescence (23). Mature hepatocytes commonly contain more than two genome copies as mononuclear or binuclear cells, and the proportion of polyploid hepatocytes increases with age and chronic liver injury (23, 24). This phenotype overlaps with several features associated with senescence, including nuclear enlargement, reduced proliferative activity, and activation of the p53/p21 pathway, but polyploidy should not be regarded as a surrogate marker of senescence (23, 25). In particular, because polyploidization can itself contribute to nuclear enlargement, the enlarged nuclear morphology described above in human MASLD tissue should not be interpreted as a senescence-specific feature in isolation. Indeed, recent evidence suggests that increased hepatocyte ploidy can buffer age-related transcriptional perturbations and, in experimental models, attenuate age-associated steatosis, indicating that polyploidization may serve an adaptive function under some conditions (26, 27). Conversely, injury-associated polyploidization can coexist with genomic damage and reduced regenerative capacity (28). Thus, in MASLD, hepatocyte ploidy is better viewed as a context-dependent cellular state that may influence the response to metabolic and genotoxic stress rather than as an intrinsically pathological feature (29). Whether specific ploidy states alter SASP composition, immune recognition, or susceptibility to immune clearance remains largely unexplored.

These observations further underscore the need for caution when identifying senescent hepatocytes *in vivo*. Cellular senescence cannot be established by p16, p21, phosphorylated histone H2AX (γ-H2AX), SA-β-gal activity, or nuclear enlargement alone (30). As illustrated by the overlap between nuclear enlargement and hepatocyte polyploidy described above, several commonly used senescence-associated features can arise through processes that are not specific to senescence. Cell-cycle inhibitors may also increase during quiescence, transient tissue repair, differentiation, or other non-proliferative states, while DNA damage and lysosomal expansion are not specific to senescence (31). The identification of senescent cells *in vivo* therefore requires convergent evidence of persistent growth arrest, stress-associated molecular or structural alterations, and functional changes (30, 32). Whenever possible, these features should be assessed using multiple markers with cell-type-specific and spatial resolution (31). This distinction is especially important in the liver because most mature hepatocytes are not actively proliferating under homeostatic conditions (30). Accordingly, confidence in hepatocyte senescence is greatest when cell-cycle arrest markers are integrated with DNA-damage, lysosomal, morphological, and functional evidence rather than interpreted independently.

These considerations make it necessary to examine not only how senescent hepatocytes are identified, but also whether they are efficiently recognized and removed by the hepatic immune system.

## 3 Immune surveillance of senescent hepatocytes

Direct evidence for immune surveillance of senescent hepatocytes in MASLD remains limited, and much of the mechanistic framework discussed below is derived from oncogene-induced liver models or from senescent cells in other tissues. These studies therefore provide proof of principle rather than direct evidence that the same mechanisms operate during metabolic liver disease. Immune surveillance limits senescent-cell persistence by coupling altered secretory and surface phenotypes to immune recognition (17). Senescent cells can release chemokines that recruit leukocytes and display stress-associated ligands that increase their susceptibility to cytotoxic attack (33). Depending on the tissue and senescence trigger, natural killer (NK) cells, T lymphocytes, monocytes, and macrophages may act sequentially or cooperatively (17). Clearance efficiency therefore reflects both the immunogenicity of the senescent cell and the functional state of the surrounding immune compartment (34). Regulatory T cells (Tregs) may also influence the local balance between immune activation and tolerance, but whether they directly regulate the recognition or clearance of senescent hepatocytes in MASLD has not been established and remains an important gap.

The clearest evidence for senescence surveillance of hepatocytes comes from an oncogene-induced mouse model rather than MASLD. In that setting, premalignant senescent hepatocytes elicited an antigen-specific type 1 helper T-cell response. CD4-positive T cells acted with recruited monocytes and monocyte-derived macrophages to remove senescent hepatocytes, whereas resident Kupffer cells were not the principal effector population (35). This work established that senescent hepatocytes can be recognized and cleared *in vivo*, but its oncogenic and antigenic context differs substantially from lipotoxic hepatocyte senescence. It should therefore be viewed as proof of principle rather than direct evidence for MASLD. Importantly, the recruited monocyte-derived macrophages described in this model should be distinguished from resident Kupffer cells, because these populations differ in origin, localization, and functional responses to hepatic injury.

Cytotoxic lymphocytes provide another potential route of surveillance. Nk cells and CD8-positive T cells can recognize stress ligands through natural killer group 2 member D (NKG2D) and eliminate susceptible senescent cells (36). In the liver, NK-cell-dependent clearance has been demonstrated for senescent activated hepatic stellate cells, although equivalent evidence for senescent hepatocytes remains limited (18). Evidence for an inhibitory HLA-E–NKG2A checkpoint is even less direct in the context of MASLD. Senescence in human dermal fibroblasts increases human leukocyte antigen E (HLA-E), which can engage the inhibitory natural killer group 2 member A (NKG2A) receptor on NK and CD8-positive T cells and reduce cytotoxic killing (37); however, whether senescent hepatocytes in MASLD employ a comparable mechanism is entirely unexplored. Nevertheless, patients with non-alcoholic fatty liver disease showed an increased circulating population of dysfunctional programmed cell death protein 1-positive NK cells, including individuals with mild fibrosis, indirectly suggesting that metabolic liver disease can weaken cytotoxic immune competence (38). This observation concerns circulating NK-cell dysfunction and should not be interpreted as direct evidence that intrahepatic NK cells fail to clear senescent hepatocytes.

Macrophages occupy a more complex position. They can remove damaged cells and coordinate adaptive responses, but are also reshaped by the lipid-rich inflammatory environment of MASLD (8). Experimental fatty liver is accompanied by remodeling of resident Kupffer-cell populations and the emergence of recruited lipid-associated macrophages with distinct transcriptional programs (39). Factors released by senescent hepatocytes can also activate macrophages rather than simply promoting silent clearance (13). Persistent exposure to lipids, cellular debris, and SASP signals may therefore shift macrophages from effective scavengers toward inflammatory or fibrogenic states. Resident Kupffer cells, recruited monocyte-derived macrophages, and lipid-associated macrophage populations should not be considered interchangeable, because their contributions to senescent-cell clearance versus inflammatory amplification may differ substantially across disease stages.

We therefore propose, as a working hypothesis rather than an established sequence, that continued generation of senescent hepatocytes may eventually exceed local clearance capacity while chronic metabolic stress simultaneously alters cytotoxic lymphocyte and macrophage function. Perforin-deficient mice accumulate more senescent cells and develop greater chronic inflammation, supporting the broader principle that impaired cytotoxicity increases senescent-cell burden (40). However, this finding does not demonstrate that the same causal sequence occurs in human MASLD. Taken together, current evidence is consistent with, but does not yet prove, the possibility that hepatocyte senescence and immune dysfunction reinforce one another during chronic metabolic injury. Continued production of senescent hepatocytes may exceed local clearance capacity, while persistent lipid and inflammatory stress progressively alters the function of cytotoxic lymphocytes and macrophages. Direct evidence for this sequence in human MASLD remains limited, however. Importantly, failure to clear senescent hepatocytes would not simply increase their number; it would also prolong their secretory activity, allowing SASP-mediated signals to reshape the surrounding hepatic microenvironment.

Testing this model directly presents several technical challenges. Senescent hepatocytes cannot be identified reliably with a single marker, newly generated senescent cells are difficult to distinguish from cells that have persisted because of failed clearance, and repeated sampling of human liver tissue is rarely feasible. In addition, ex vivo tissue processing can alter immune-cell phenotype and disrupt the spatial relationships required to infer cell–cell interactions. Progress will therefore require combinations of spatial profiling, multiplex senescence markers, longitudinal or lineage-tracing approaches in experimental models, and functional assays that directly measure immune recognition and killing of senescent hepatocytes.

## 4 SASP-mediated remodeling of the fibrotic niche

The SASP is not a uniform collection of inflammatory mediators, but a context-dependent communication program shaped by the senescence trigger, cell lineage, duration of arrest, and surrounding tissue (see **Figure 1**) (41). Accordingly, the senescent hepatocyte secretome is better considered as an interacting network rather than a fixed set of universally inflammatory or profibrotic molecules. Senescent hepatocytes may release cytokines, chemokines, growth factors, proteases, lipids, and extracellular vesicles, thereby influencing cells that are not themselves senescent (42). A transient SASP can support immune recruitment and tissue repair (43). In chronically injured liver, however, continued production of senescent hepatocytes and incomplete immune clearance can prolong these signals and convert an initially adaptive response into maladaptive remodeling (44).

**Figure 1:**
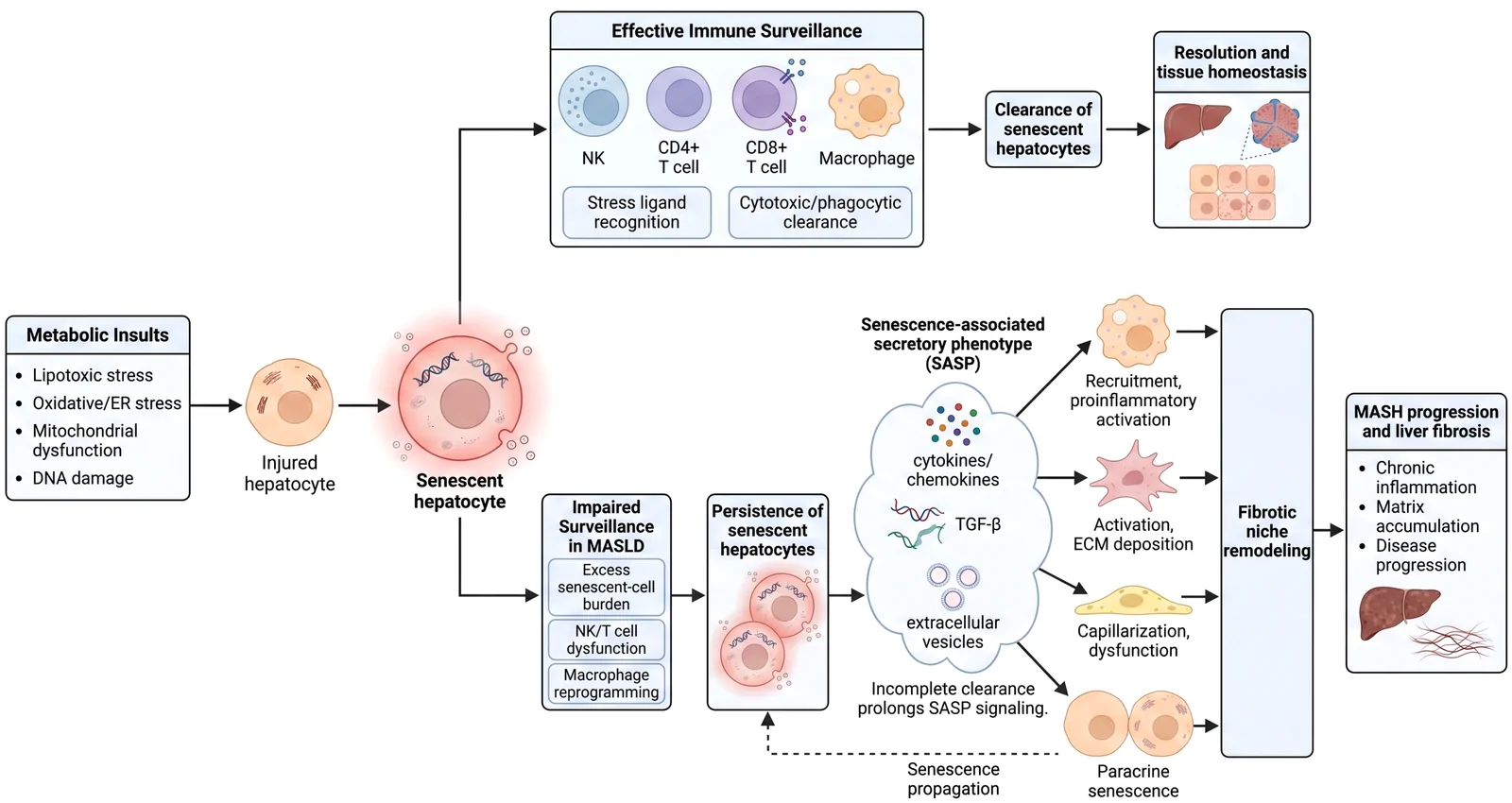
Impaired immune surveillance of senescent hepatocytes and SASP-mediated remodeling of the fibrotic niche in MASLD. Persistent metabolic insults, including lipotoxic stress, oxidative and endoplasmic reticulum stress, mitochondrial dysfunction, and DNA damage, promote hepatocyte injury and senescence. Under effective immune surveillance, natural killer cells, CD4-positive and CD8-positive T cells, and macrophages can participate in the recognition and clearance of senescent hepatocytes, thereby favoring resolution of inflammation and restoration of tissue homeostasis. In metabolic dysfunction-associated steatotic liver disease (MASLD), however, excessive generation of senescent hepatocytes, dysfunction of cytotoxic lymphocytes, and macrophage reprogramming may compromise effective clearance and permit senescent cells to persist. Sustained senescence-associated secretory phenotype (SASP) signaling subsequently reshapes the hepatic microenvironment by promoting macrophage recruitment and proinflammatory activation, hepatic stellate cell activation and extracellular matrix deposition, liver sinusoidal endothelial cell capillarization and dysfunction, and paracrine senescence in neighboring hepatocytes. The latter may further expand the senescent-cell pool, establishing a self-reinforcing cycle of persistent SASP signaling. Together, these processes contribute to fibrotic niche remodeling, chronic inflammation, matrix accumulation, and progression toward metabolic dysfunction-associated steatohepatitis (MASH) and liver fibrosis. Created by BioRender (Agreement number: CG2A3LO9V9).

Macrophages are among the earliest cells affected by the hepatocyte secretome. Conditioned medium from stress-induced senescent human hepatocytes promotes macrophage migration, indicating that the hepatocyte SASP can recruit myeloid cells to sites of injury (45). More recent experiments showed that medium from hepatocytes expressing a senescent hepatocyte gene signature increased proliferative and proinflammatory programs in macrophages (13). Recruitment alone is therefore not equivalent to successful clearance. When macrophages are repeatedly exposed to lipids, cellular debris, and SASP factors, they may remain inflammatory or acquire fibrogenic functions rather than resolving injury (46). This creates a feed-forward circuit in which senescent hepatocytes attract immune cells, yet the recruited or resident macrophages fail to remove them efficiently and instead amplify local cytokine signaling (47). These observations raise the possibility of a feed-forward interaction in which persistent senescent hepatocytes recruit or reprogram macrophages, while ineffective clearance allows inflammatory signaling to continue. However, this proposed circuit has not been traced directly from senescent-hepatocyte generation through macrophage dysfunction to fibrosis in a single MASLD model and should therefore be regarded as a mechanistic hypothesis rather than an established sequence.

Hepatic stellate cells (HSCs) provide the principal cellular route from chronic inflammation to scar formation (48). In human MASLD tissue, hepatocyte p16 expression correlates with HSC activation and fibrosis severity (49). Because p16 alone is not specific for cellular senescence, this association should be interpreted together with the multi-marker considerations described above rather than as direct evidence of a causal senescent-hepatocyte–HSC axis. Conditioned medium from senescent hepatocytes also alters HSC transcription and increases inflammatory and fibrogenic responses *in vitro (*50). These findings support a paracrine link, although they do not establish that a single SASP component is responsible (49). Transforming growth factor beta (TGF-β), interleukin-6 (IL-6), platelet-derived growth factors (PDGFs), and extracellular-vesicle cargo may contribute in a stimulus-dependent manner (49). Importantly, the secretome should be considered as an interacting network rather than a fixed list of universally profibrotic molecules.

Senescent hepatocytes also affect liver sinusoidal endothelial cells (LSECs) and neighboring parenchymal cells. In a recent MASLD-focused study, senescent-hepatocyte conditioned medium impaired LSEC functional markers and tube formation, consistent with capillarization, while inducing secondary senescence and renewed SASP expression in non-senescent hepatocytes (13). Endothelial capillarization can disturb sinusoidal exchange and alter leukocyte trafficking, whereas paracrine senescence expands the population capable of maintaining inflammatory signals. The same study identified growth differentiation factor 15 (GDF15) as part of the senescent hepatocyte communication network, but its net effect remains uncertain because GDF15 has context-dependent metabolic and stress-response functions (13). GDF15 is therefore included here not as an established profibrotic mediator, but as an example of a candidate SASP-associated signal whose causal contribution to macrophage, endothelial, or stellate-cell remodeling remains to be determined.

Taken together, these observations are consistent with, but do not yet establish, a self-reinforcing model in which persistent senescent hepatocytes progressively reshape the immune and stromal microenvironment. Their secretome may recruit and reprogram macrophages, activate hepatic stellate cells, disturb sinusoidal endothelial homeostasis, and propagate senescence to neighboring hepatocytes (51, 52). If immune clearance remains effective, this response may remain transient (51). If clearance is impaired, however, prolonged SASP signaling may stabilize an inflammatory and fibrogenic niche (51). This distinction is also therapeutically important, because intervention could theoretically target the senescent cell itself, its secretory program, or the immune mechanisms responsible for its removal.

## 5 Therapeutic strategies targeting hepatocyte senescence

Senescence-directed treatment may involve eliminating senescent cells, limiting their harmful secretory activity, or redirecting immune clearance (20). Senolytics selectively induce death in senescent cells, although their activity depends strongly on cell lineage and the stress responsible for senescence (53). In a recent chemical screen, the thiosemicarbazone DpC preferentially eliminated senescent hepatocytes. Treatment reduced hepatic senescence, inflammation, steatosis, and fibrosis in male mice with diet-induced MASH (13). These results contrast with those obtained using dasatinib plus quercetin (D+Q) or fisetin, which failed to reduce liver senescence, inflammation, or injury in a choline-deficient Western-diet model (54). This discrepancy may reflect differences in the senescent-cell state, disease model, treatment timing, and, importantly, the selectivity of individual compounds for senescent hepatocytes. In the DpC study, chemical screening showed that DpC preferentially targeted senescent hepatocytes through a copper-dependent pro-apoptotic mechanism, whereas commonly used senolytics such as dasatinib showed little selectivity for the same senescent hepatocyte population (13). Senolytic efficacy should therefore not be regarded as a class effect. Sex may represent an additional source of variability. A recent study in female mice with diet-induced MASLD found that D+Q modestly altered selected hepatic senescence- and inflammation-related measures but produced only limited improvement in liver pathology, despite clearer effects on reproductive outcomes (55). These findings suggest that both sex and tissue context should be considered when evaluating senolytic responses. Candidate compounds need to be evaluated against the relevant hepatocyte senotype and in models that reflect the metabolic and fibrotic features of human disease.

Senomorphics offer a less destructive alternative by modifying the SASP without directly killing the arrested cell (56). Inhibition of the mechanistic target of rapamycin pathway can suppress SASP production by reducing the translation of mitogen-activated protein kinase-activated protein kinase 2 (57). Thus, the mechanistic target of rapamycin (mTOR)–MAPKAPK2 axis represents one defined pathway through which SASP output can be modulated. This approach is not necessarily beneficial in every setting, because selected SASP components recruit immune cells required for senescent-cell removal (58). Broad or prolonged SASP inhibition could consequently reduce inflammation while allowing dysfunctional hepatocytes to persist (58). Rather than globally suppressing the SASP, a more selective strategy would be to inhibit sustained inflammatory or fibrogenic signaling while preserving chemotactic signals required for immune surveillance and tissue repair; however, hepatocyte-specific evidence supporting such selective senomorphic approaches in MASLD remains limited.

Immune-based senolysis provides preclinical proof of principle that senescence surveillance can be therapeutically redirected. Chimeric antigen receptor (CAR) T cells targeting the urokinase-type plasminogen activator receptor (uPAR) eliminated senescent cells and reduced diet-induced steatohepatitis-associated fibrosis in mice (59). However, these findings should not be interpreted as evidence for selective elimination of senescent hepatocytes, because uPAR is not restricted to this cell population. Nevertheless, uPAR is not exclusive to senescent hepatocytes, and the persistence and systemic activity of engineered immune cells raise safety concerns (60). Similar caution applies to enhancing NK-cell cytotoxicity or macrophage phagocytosis, because these cells also regulate hepatocyte injury, inflammation, and tissue repair (61). Accordingly, therapeutic restoration of NK-cell or macrophage-mediated senescence surveillance in MASLD remains largely conceptual until hepatocyte-selective recognition mechanisms and safety boundaries are established.

Translation will require cell-type-specific markers, confirmation of durable senescence, and evidence that cell removal improves rather than restricts regeneration. As discussed in Section 2, commonly used features such as p16, p21, γ-H2AX, SA-β-gal activity, and nuclear enlargement are not individually sufficient to define a senescent hepatocyte. Therapeutic studies should therefore use convergent marker panels or hepatocyte-specific senescence signatures to confirm that the targeted population is truly senescent. Disease stage, sex, metabolic context, and the contrasting effects of hepatocyte and hepatic stellate-cell senescence must also be considered. Rather than indiscriminately suppressing senescence, future therapies should combine patient stratification with temporally controlled targeting of pathogenic senescent hepatocytes while preserving beneficial repair programs.

## 6 From association to mechanism

Current evidence links hepatocyte senescence to MASLD progression, but the mechanisms that determine whether senescent hepatocytes are efficiently cleared or persist within the liver remain incompletely defined. One possibility is that persistent senescent hepatocytes acquire an immune-evasive phenotype (37). The HLA-E–NKG2A axis is a plausible candidate because HLA-E can suppress natural killer (NK)-positive and CD8-positive T-cell cytotoxicity in other senescent-cell contexts, while dysfunctional cytotoxic lymphocyte populations have been described in metabolic liver disease (37, 62). Whether this pathway contributes to hepatocyte persistence in MASLD could be examined by spatial profiling of human liver tissue and by co-culture systems combining lipotoxicity-induced senescent hepatocytes with NK or CD8-positive T cells, followed by HLA-E or NKG2A manipulation (63).

A second question is whether specific components of the senescent hepatocyte secretome actively drive fibrotic remodeling or primarily reflect cellular stress (64). GDF15 represents one candidate because it has been associated with the senescent hepatocyte signature and MASLD severity, although its functional role remains uncertain (65). Hepatocyte-specific loss-of-function, neutralization, or rescue experiments in diet-induced MASH models could help determine whether GDF15 directly contributes to macrophage reprogramming, endothelial dysfunction, stellate-cell activation, or paracrine senescence (66).

Finally, the pathogenic significance of hepatocyte senescence is likely to depend on timing (67). Transient senescence may facilitate damage containment and immune clearance, whereas prolonged persistence may sustain SASP signaling and impair regeneration (67, 68). Longitudinal lineage-tracing approaches, combined with stage-specific depletion or immune manipulation, could clarify when hepatocyte senescence shifts from an adaptive response to a maladaptive state (15). Defining this transition may be more informative than simply quantifying senescent-cell abundance and could help identify the disease stages and molecular targets most suitable for senescence-directed therapy (15). Together, these candidate mechanisms provide a framework for moving from descriptive associations toward causal investigation of senescent hepatocyte persistence in MASLD (see **Table 1**).

**Table 1:** Key cellular and molecular determinants of senescent hepatocyte persistence in MASLD.

Candidate determinant	Proposed role	Current evidence	Major unresolved question	Experimental approach
HLA-E–NKG2A signaling	May restrain NK- and CD8-positive T-cell cytotoxicity and thereby favor persistence of senescent cells.	HLA-E-mediated inhibition of cytotoxic lymphocytes has been demonstrated in senescent non-hepatic cells; dysfunctional cytotoxic lymphocyte populations have also been reported in metabolic liver disease.	Whether senescent hepatocytes activate the HLA-E–NKG2A checkpoint in human MASLD remains unknown.	Spatial profiling of HLA-E and NKG2A in human MASLD liver; senescent hepatocyte–lymphocyte co-culture with HLA-E knockdown or NKG2A blockade.
NKG2D and stress-associated ligands	May promote recognition and elimination of stressed or senescent cells by NK and CD8-positive T cells.	NKG2D-dependent immune surveillance has been implicated in senescent-cell clearance, although direct evidence in senescent hepatocytes is limited.	Which NKG2D ligands are expressed by senescent hepatocytes, and whether their expression changes during MASLD progression, are unclear.	Multiplex tissue imaging; ligand profiling of senescent hepatocytes; genetic or antibody-based perturbation followed by cytotoxicity assays.
Macrophage state	May determine whether recruited myeloid cells support senescent-cell clearance or instead amplify inflammatory and fibrogenic signaling.	MASLD is accompanied by Kupffer-cell remodeling and recruitment of macrophage populations with distinct transcriptional programs; senescent hepatocyte-derived factors can promote macrophage migration and inflammatory activation.	Whether macrophage dysfunction precedes senescent-cell accumulation or develops secondary to persistent lipid and SASP exposure remains unresolved.	Spatial transcriptomics; lineage tracing; macrophage depletion or reconstitution; hepatocyte–macrophage co-culture.
GDF15	May participate in communication between senescent hepatocytes and surrounding immune, endothelial, or fibrogenic cells.	GDF15 has been identified within the senescent hepatocyte communication network and is associated with the senescent hepatocyte signature, but its net biological effect remains context dependent.	Whether GDF15 is a causal mediator of tissue remodeling or a compensatory stress-response signal is uncertain.	Hepatocyte-specific loss-of-function; neutralization; recombinant GDF15 rescue; assessment of macrophages, LSECs, HSCs, and paracrine senescence.
Hepatocyte polyploidy	May modify stress tolerance, proliferative potential, and the cellular response to metabolic or genotoxic injury.	Polyploidy is a common feature of mature hepatocytes and increases with ageing; experimental evidence suggests that its effects can be adaptive or detrimental depending on context.	Whether hepatocyte ploidy influences senescence induction, SASP composition, immune recognition, or immune-mediated clearance in MASLD is largely unknown.	Ploidy-resolved transcriptomic or spatial profiling; lineage tracing; experimental manipulation of hepatocyte ploidy followed by senescence and immune-surveillance analyses.
Duration and stage of senescence	May determine whether senescence remains a transient damage-containment response or develops into a persistent maladaptive state.	Transient senescence can support damage containment and immune-mediated removal, whereas prolonged senescent-cell persistence may sustain SASP signaling and impair regeneration.	When, during progression from steatosis to MASH and fibrosis, hepatocyte senescence becomes predominantly pathogenic remains undefined.	Longitudinal lineage tracing; stage-specific senescent-cell depletion; temporally controlled immune manipulation during disease progression and regression.

CD8, cluster of differentiation 8; GDF15, growth differentiation factor 15; HLA-E, human leukocyte antigen E; HSCs, hepatic stellate cells; LSECs, liver sinusoidal endothelial cells; MASLD, metabolic dysfunction-associated steatotic liver disease; MASH, metabolic dysfunction-associated steatohepatitis; NK, natural killer; NKG2A, natural killer group 2 member A; NKG2D, natural killer group 2 member D; SASP, senescence-associated secretory phenotype.

The proposed roles listed here should be interpreted as mechanistic hypotheses rather than established pathways in human MASLD. In particular, direct evidence for HLA-E–NKG2A-mediated immune evasion, ploidy-dependent immune surveillance, and the temporal transition from adaptive to pathogenic hepatocyte senescence in human MASLD remains limited.

## 7 Discussion

This Review evaluates the hypothesis that impaired immune surveillance may contribute to the persistence of senescent hepatocytes and thereby link chronic metabolic stress to inflammatory and fibrotic remodeling in MASLD. However, senescence should not be regarded as uniformly harmful. Transient senescence may restrict the proliferation of damaged cells and facilitate immune-mediated clearance, whereas prolonged persistence can impair regeneration and sustain inflammatory signaling. Its biological consequences also vary across hepatic cell types: persistent hepatocyte senescence is generally associated with metabolic dysfunction and fibrogenic remodeling, while senescence of activated hepatic stellate cells may limit proliferation and extracellular matrix production in certain settings. This heterogeneity, together with the lack of a single specific senescence marker, remains an important limitation when interpreting both experimental and human studies. Moreover, the relative contributions of continued hepatocyte injury, defective immune clearance, altered SASP signaling, and cell-type-specific senescence are likely to vary across disease stages and have not yet been quantitatively resolved.

The available evidence further suggests that senescent-cell accumulation in MASLD may reflect both continued hepatocyte injury and insufficient immune-mediated clearance, although direct longitudinal evidence for surveillance failure in human disease remains limited. Persistent SASP signaling may subsequently reshape the hepatic microenvironment by promoting macrophage reprogramming, stellate-cell activation, endothelial dysfunction, and paracrine senescence, thereby reinforcing inflammation and fibrosis. Future studies should therefore address the specific barriers identified in this Review by combining multiplex spatial profiling of human liver tissue with multi-marker senescence identification, longitudinal or lineage-tracing approaches, and functional assays of immune recognition and killing. Such designs will be particularly important for distinguishing newly generated senescent hepatocytes from cells that persist because of failed clearance and for determining whether proposed immune checkpoints are causally involved. Taken together, the key issue is not simply whether senescent hepatocytes accumulate, but when a transient stress response becomes a persistent state that escapes effective clearance and drives maladaptive tissue remodeling. At present, this transition remains a mechanistic model rather than a demonstrated sequence in human MASLD, and the therapeutic value of targeting it therefore remains to be established. As a narrative Mini Review, the present synthesis was guided by mechanistic relevance rather than a formal systematic-review protocol and may therefore be subject to literature-selection bias and incomplete coverage of the available evidence. A decisive test of the central hypothesis would require showing that selective disruption or restoration of a defined senescent-hepatocyte immune-recognition pathway alters senescent-cell persistence and downstream fibrosis without simply changing the rate at which hepatocytes enter senescence.
